# Integrin α5β1, the Fibronectin Receptor, as a Pertinent Therapeutic Target in Solid Tumors

**DOI:** 10.3390/cancers5010027

**Published:** 2013-01-15

**Authors:** Florence Schaffner, Anne Marie Ray, Monique Dontenwill

**Affiliations:** UMR 7213 CNRS, Laboratoire de Biophotonique et Pharmacologie, Tumoral signaling and therapeutic targets, Université de Strasbourg, Faculté de Pharmacie, 67401 Illkirch, France; E-Mails: florence.schaffner@unistra.fr (F.S.); amray@unistra.fr (A.M.R.)

**Keywords:** α5β1, integrin, fibronectin receptor, solid tumors, angiogenesis, antagonists

## Abstract

Integrins are transmembrane heterodimeric proteins sensing the cell microenvironment and modulating numerous signalling pathways. Changes in integrin expression between normal and tumoral cells support involvement of specific integrins in tumor progression and aggressiveness. This review highlights the current knowledge about α5β1 integrin, also called the fibronectin receptor, in solid tumors. We summarize data showing that α5β1 integrin is a pertinent therapeutic target expressed by tumoral neovessels and tumoral cells. Although mainly evaluated in preclinical models, α5β1 integrin merits interest in particular in colon, breast, ovarian, lung and brain tumors where its overexpression is associated with a poor prognosis for patients. Specific α5β1 integrin antagonists will be listed that may represent new potential therapeutic agents to fight defined subpopulations of particularly aggressive tumors.

## 1. Introduction

In recent years, integrins have attracted increasing interest for their potential to act as tumor therapeutic targets [1,2]. First recognized as cell adhesion molecules and receptors for the extracellular matrix (ECM), it is now widely acknowledged that integrins act as true receptors regulating intracellular signaling and cellular responses including migration, proliferation and differentiation [3]. Integrins are αβ protein heterodimers whose non covalent association defines the specificity of adhesion to particular components of the ECM or other proteins (immunoglobulin superfamily molecules, plasma proteins, VCAM1, *etc*.). In mammals, 18 α subunits and eight β subunits form a large family of about 24 αβ integrins, which bind to tissue and organ specific ligands. Regulating the crosstalk between cells and their surrounding microenvironment, integrins are particularly relevant in different key aspects of tumor progression. Depending on the tumor types, the expression of specific integrins differs between tumoral tissues and their corresponding healthy tissues. Integrins are overexpressed in cancer cells impacting proliferation, survival, resistance to therapies and tumor recurrence. In addition, an important role in tumor angiogenesis has been highlighted for several integrins including α5β1 and αvβ3/β5. Their overexpression on neo-vessels during the tumor angiogenic switch led to the suggestion of new anti-angiogenic therapies [4]. Cilengitide, a specific antagonist for αvβ3/β5 integrins is currently in clinical trials for the treatment of different tumors as for example the highly vascularized brain tumor glioblastoma [5]. Different recent reviews have already summarized the current knowledge about integrin structure, activation and signaling as well as integrin implication as therapeutic targets in cancer and/or angiogenesis [2,3,4,6,7,8,9]. The goal of this review is to focus on α5β1 integrin, also called the fibronectin receptor, as an emerging therapeutic target in different solid tumors. The role of α5β1 integrin in cancer has been somewhat controversial with data suggesting tumor suppressive effects while others are in favor of a protumoral behavior. In the last decade, the relationship between α5β1 integrin expression on tumors and patient survival has become increasingly recognized in several cancers. In this article we provide an overview of the implication of α5β1 integrin in tumor angiogenesis, and solid tumor aggressiveness and we list the currently available α5β1 integrin antagonists.

## 2. Generalities on α5β1 Integrin

Integrins are composed of an extracellular domain, a transmembrane domain and a short cytoplasmic tail. The α subunit extracellular domain has a 7-bladed β propeller connected to a thigh and two calf domains. In addition nine of the α subunits have an I domain that contains the metal ion-dependent adherent site (MIDAS), crucial for ligand binding [10]. The α subunit is responsible for the high specificity of α5β1 integrin for fibronectin. The combination of a primary interaction with the RGD site with a secondary interaction with the synergy site of fibronectin ensures both high affinity and specificity of α5β1 integrin for fibronectin [11,12]. A recent crystallography structure of α5β1 integrin in complex with a RGD-peptide has indicated that interaction of this integrin with the synergy site comes from the residue Asp 154 in the α5 subunit and also that Ca^2+^ is an important cation for fibronectin binding [13]. The β subunit extracellular part is composed of a hybrid domain, a betaI domain (with a MIDAS structure), a plexin/semaphorin/integrin domain and four EGF like domains [14].

Crystal structures have revealed that β3 integrins occur in three possible states: low, intermediate and high affinity for its ligand [15,16]. Activation of integrin either by binding to its ligand (outside-in signaling) or by binding of an activator protein like talin to its cytoplasmic tail (inside-out signaling) result in a change in conformation from a bent (low affinity) to an extended (high affinity). At the proximal site of the transmembrane domain GFFKR, residues, extremely conserved in the α subunit, and hydrophobic membrane-proximal residues in the β subunit, are involved in releasing the integrin from an inactive to an active conformation, with unbound cytoplasmic tails available for signal transduction [7,17]. Integrins do not have enzymatic activity therefore signal transduction is performed by proteins recruited to the cytoplasmic tail such as FAK, src, and talin [1,7]. Length and intensity of the signal is regulated in part by specific negative regulators proteins such as ICAP1 or sharpin [18]. Integrin α5β1 has specific regulator such as nischarin [19] and GIPC1 [20]. In addition to the recruitment of signaling inhibitors, integrin trafficking can regulate signaling [21]. After activation, α5β1 integrin can either be ubiquitinated and degraded in lysosome or get recycled rapidly to the plasma membrane via the early endosome pathway [21]. Ubiquitination and degradation in lysosome of α5β1 integrin is important for proper fibroblast migration on fibronectin [22]. Integrins can also be internalized via clathrin dependent or independent pathway [21]. Caveolar internalization of α5β1 integrin and fibronectin promotes matrix turnover [23]. Interestingly, it seems that caveolin-1 is capable of internalizing α5β1 integrin without fibronectin, thereby regulating the amount of the fibronectin receptor available on the cell surface. This type of regulation was also shown on endothelial cell with internalization of active or inactive α5β1 integrin by two different pathways [20]. Rapid recycling of β1 integrin to the plasma membrane through early endosome is regulated by Rab5-related GTPase Rab21 binding to a region close to the GFFKR motif [24]. Other small GTPase protein regulates α5β1 integrin like Rab25 that promotes invasion in cancer cells [25,26,27]. Recent data also point to a role of α5β1 integrin in mechanosensing [28,29]. In response to mechanical forces, α5β1 integrin switches between relaxed and tensioned states which allow strong adhesion and downstream signals [30,31]. The synergy site in fibronectin is required to form the tensioned bonds although the relaxed bonds only involve the RGD site [31]. Mechanical stimulation of α5β1 integrin enhances cancer cell invasion [32,33]. In summary, integrin bidirectional signaling regulates several processes such as migration, invasion, proliferation and survival specific to each cell types and is a major regulator of cancer progression which will be discussed next [1,6,8].

## 3. Integrin α5β1 and Angiogenesis

Angiogenesis is a key physiological and pathological process that is regulated in part by integrins. Integrins are expressed by endothelial cells, smooth muscle cells and cellular components of the blood such as platelets, monocytes, lymphocytes *etc*. for example [4]. Among the large integrin family, α4, α5, αv or β1 integrin subunits were shown to be required for vasculo- and angiogenesis during development [34,35,36]. Yet, each of these integrins have specific function: α4 knock out mice display an abnormal retention of hematopoietic stem cells in the bone marrow niche and cardiac defect, αv knock out mice show predominantly hemorrhage in the brain and intestine resulting in death from mid-gestation to perinatal, and β1 knock out specifically in endothelial cells resulting in the most severe phenotype with vascular remodeling defects caused by adhesion and migration alteration and reduced survival of endothelial cells. Homozygous deletion of α5 integrin subunit results in embryonic death at around E 10.5 due to defects in posterior trunk development (defects in neural tube and somites), and angiogenesis [37,38]. The α5 null embryos and the extraembryonic vessels display defects resulting in leakage of blood cells. Angiogenesis abnormalities are also observed inmice deficient in fibronectin, the major ligand for α5β1 integrin, although lethality occurs earlier at E9.5 [39]. To further understand the mechanism regulated by α5 integrin subunit in angiogenesis, conditional knock out were designed where α5^flox/flox^ mice were crossed with Tie2-Cre mice. The resulting mice do not express a5 integrin subunit on endothelial and hematopoietic cells. Surprisingly the embryos developed normally and do not harbor angiogenesis defects [40]. Analysis of adhesion of α5 null endothelial cells shows that αv is present to focal adhesion points and therefore compensates for the lack of α5. Double knock out of α5 and αv in endothelial cells results in abnormal vessel remodeling and heart defects in most of the embryos by E14.5. These results highlight the importance of specific integrins in developmental angiogenesis but also that compensation mechanisms by other integrins exist in order to complete angiogenesis. These mechanisms are not only observed during development but also for example in adult hypoxic brain endothelial cells [41].The compensation mechanisms may play crucial role and should be taken into consideration when analyzing the results of integrin targeting therapy. 

In addition to its direct role in angiogenesis, integrin α5β1 also regulates angiogenic signals by binding with different partner such as endostatin [42], VEGFR-1 [43], Angiopoietin-2 and Tie-2 [44]. Interestingly, mature vessels present very low level of α5 integrin subunit (with the exception of hepatic sinusoid and high-endothelial venules in lymph nodes) whereas tumor vasculature or neovessels in the cornea express high level of α5 [45,46,47,48]. Injection of a specific monoclonal anti-α5 antibody in several murine cancer models shows that α5 integrin subunitis expressed on the luminal side of the tumor vasculature and thereby directly accessible for potential anti-α5 agents [49]. Integrin subunit α5 expression in endothelial cells is regulated by several angiogenic factors such as FGF, TNFα or IL8, but not VEGF [45]. In turn, activation of α5β1 on endothelial cells by attachment to fibronectin results in the transcription of a gene repertoire related to angiogenesis (HB-EGF, IL8, CXCL1), adhesion (VCAM, E-selectin), signal transduction (RICK, NFκb) and coagulation (TF) [50]. Once expressed, α5 integrin subunit promotes survival signals in angiogenic endothelial cells and blocks apoptotic signals independently of attachment to matrix *in vitro* and *in vivo* [51]. Therefore, blocking α5 integrin subunit with a small peptide or an antibody results in anti-angiogenic effects and reduced tumor growth by integrin-mediated death pathway [45,52,53]. Due to its unambiguous role in angiogenesis, α5β1 integrin has become a target for anti-angiogenesis therapy.

## 4. Integrin α5β1 in Solid Tumors

### 4.1. Colon Tumors

The controversy about α5β1 integrin as a tumor suppressor rather than a protumoral integrin mainly arose from data obtained in a colon cancer cell line, HT29. Studies showed that *de novo* expression of α5 integrin subunit in HT29 cells results in cell growth arrest *in vitro* and decreased tumorigenicity *in vivo*. Cell growth arrest was reversed by ligation of α5β1 integrin to fibronectin [54]. Interestingly, α5β1-expressing HT29 cells were shown to resist to serum deprivation-induced apoptosis [55]. The tumor suppressive function of α5β1 integrin in HT29 cells was confirmed in another study [56] and a strong inhibitory action of this integrin on lung colonization and metastasis was also reported [57]. These results were challenged when subgroups of colon cancer cell lines were examined according to their differentiation status [58]. It was shown that integrin α5β1 level was increased in the poorly differentiated group in relationship with an increased capacity to form tumors in nude mice [59]. In accordance with these results, three well-established colon cancer cell lines, KM20, KM12C and KML4A, treated with an anti-α5 integrin inhibitory antibody, increased their apoptosis rate [60]. It was recently found that 19% of colon carcinoma, over 94 tumors examined, expressed α5β1 integrin at the protein level and in these tumors the labeling concerned only a fraction of neoplastic epithelial cells [61]. Interestingly, acquisition of α5β1 integrin was correlated with ADAM-15 down-regulation and poor prognosis [61]. In line with this, hypoxia was shown to increase α5 integrin subunit at the mRNA level and this increase was more prominent in Duke stage C and D patients than in Duke stage A and B patients suggesting that the transcription increases along with the progression of colon cancer [62]. Upregulation of α5 integrin subunit gene transcription in colon cancer cells is under the control of PTHrP [63] or ZEB2 [64] and leads to an upregulation of cell invasion during epithelial-mesenchymal transition. Activation of α5β1 integrin and corresponding signaling pathways by P-selectin and the human carcinoembryonic antigen (CEA) was also reported in colon carcinoma cells [65,66]. Suppression of α5β1 integrin activity by lunasin, a peptide isolated from soybean and having an RGD motif, potentiates the effect of oxaliplatin thus preventing outgrowth of colon cancer metastasis [67]. 

### 4.2. Ovarian Tumors

Peritoneal dissemination is an important step in ovarian cancer progression to invasion and metastasis. It was first reported that fibronectin secreted by peritoneal tissue activates α5β1 integrin on ovarian cancer cells to stimulate their invasiveness through an increase of MMP-9 activity [68]. α5β1 integrin regulates the formation of ovarian carcinoma multicellular spheroids, an *in vitro* model of micrometastasis [69], and partially mediates adhesion to mesothelial cell monolayer of patient-derived ascites spheroids [70]. Many human ovarian cancer cell lines express α5β1 integrin and their binding to mouse peritoneal wall preparation was impaired specifically by anti α5β1 integrin antibodies or endostatin which is a ligand for α5β1 integrin [71,72]. Kallikrein-related peptidases (KLK) are serine proteases often upregulated in ovarian carcinoma. KLK7 overexpression correlates with formation of large compact spheroids, chemoresistance and poor outcome in clinical settings. Interestingly enhanced expression of KLK7 in ovarian cancer cell lines and clinical samples was associated with enhanced expression of α5β1 integrin [73] suggesting that α5β1 integrin participates to the poor outcome of patients. The hypothesis of α5β1 integrin as a prognostic marker in ovarian tumors is confirmed by other data including large cohorts of patients [74,75]. In one of this study [74], α5β1 integrin expression was inversely correlated with E-cadherin expression and was shown to be implicated in adhesion of tumor cells to the peritoneal cavity and metastasis. Inhibition of α5β1 integrin by specific antibodies led to the suppression of intra-peritoneal tumor spread and increased survival in two xenograft models of ovarian cancer. In fact fibronectin/α5β1 integrin interaction on ovarian cancer cells activates the oncogene cMet and provides key mitogenic-signalling pathways to the cells [76]. Adrenomedullin also upregulates α5β1 integrin in ovarian tumors and patients with high adrenomedullin expression showed a higher incidence of metastasis and poor outcomes, indirectly further suggesting a role of α5β1 integrin in the aggressiveness of ovarian tumors [77]. An overview of integrin inhibitors as therapeutic agents for ovarian cancer has been published very recently [78].

### 4.3. Breast Tumors

Similarly to what was shown in colon cancer cells, the first data concerning α5β1 integrin in breast tumor cells were in favor of its tumor suppressive effect. It was reported that treatment of the highly invasive breast carcinoma cell line MDA-MB-435 (which has been further classified as a melanoma cell line) with Maspin suppressed their invasive phenotype through an increased expression of α5β1 integrin at the mRNA and protein level [79]. Subsequent data however challenged this view as they demonstrated a proinvasive role of α5β1 integrin in breast cancer cells [80,81,82]. The oncogene ERBB2, strongly associated with metastatic disease and poor prognosis, drives the transcriptional upregulation of α5β1 integrin in mammary adenocarcinoma promoting tumor cell survival under adverse conditions and invasive capacity [80,83]. In a subset of breast cancers, overexpression of Steroid Receptor Coactivator-1 (SRC-1) was associated with an upregulation of α5β1 integrin and promotion of α5β1 integrin-dependent cell adhesion and migration [84]. Inverse relationship between α5β1 integrin expression and tumor suppressors expression such as nischarin [85], metastasis suppressors such as Nm23 [86] or epithelial cell-cell adhesion marker such as E-cadherin [87] were reported and associated with impact on breast cell tumorigenic potential. Loss of E-cadherin was also achieved through stimulation of breast cancer cells by angiopoietin-2 which stimulated cell migration through an α5β1 integrin-dependent way [88]. Data also showed that α5β1 integrin controls invasion of breast cancer cells by modulation of MMP-1 [81] and MMP-2 collagenase activity [89]. α5 integrin subunit mRNA was weakly expressed in normal tissues and more strongly expressed in breast cancer specimens [90] and elevated α5 integrin subunit gene expression was associated with decreased long term survival in one cohort of patients with breast cancer [91] but not in two other cohorts [92]. Interestingly, while α5 integrin subunit was proposed to be positively involved in lung metastasis of breast tumors in humans [85], the opposite effect was described for mouse breast tumor cells [93]. Finally, radiotherapy was shown to increase α5β1 integrin expression level in 3D culture breast tumor cells and combined cell treatment with ionizing radiation and antagonists of α5β1 integrin triggered apoptosis [91].

### 4.4. Lung Tumors

Tobacco is the major risk factor for lung tumors. The main tobacco alkaloid nicotine stimulates lung cancer cell proliferation by the induction of fibronectin that led to activation of the α5β1 integrin. In non small cell lung cancer, α5β1 integrin overexpression at the mRNA [94,95] or protein [96,97] level was negatively associated with patient survival. Interestingly, α5β1 integrin expression could differentiate between adenocarcinoma and squamous cell carcinoma of the lung [94]. α5β1 integrin expression was more frequent in tumors with lymph node metastasis than in those without metastasis [96]. Fibronectin-α5β1 integrin signaling has been studied and implicated in lung cancer progression [98,99,100] and reviewed in [101]. The PI3K/AKT/mTOR pathway is a key mediator of fibronectin-integrin effects on proliferation [99]. Extracellular matrix proteins including fibronectin were shown to protect lung cancer cells from apoptosis through β1 integrin activation [102,103,104,105] thus explaining drug resistance of lung cancer cells.

### 4.5. Glioma

The α5β1 integrin is expressed at significantly higher level in glioblastoma (the most aggressive glioma) than in adjacent normal brain tissue suggesting that it might play a role in the development or the progression of glioma [106]. α5β1 integrin is commonly expressed in a perinecrotic or perivascular pattern in glioblastoma [107]. α5 integrin subunit mRNA level is under the control of the transcription factor ETS-1 and its expression is related to the grade of glioma, with the highest expression in glioblastoma [108]. We [109,110] and others [111] confirmed recently these data in larger cohorts of patients and showed that a high expression of α5β1 integrin is associated to a worse prognostic for patients with glioma. We also demonstrated that α5β1 integrin expression is under the negative control of caveolin-1 and positive control of TGFβR in a subset of glioma tumors [110,112]. By the use of specific non peptidic antagonists of α5β1 integrin, its role in proliferation, migration, invasion and resistance to chemotherapy was highlighted in different glioma cell lines [109,113,114]. Interaction of MMP-2 with α5β1 integrin was shown to regulate the IL-6/STAT3 survival signaling in glioma [115]. The expression of the DNA repair protein, O6-Methylguanine-DNA Methyltransferase (MGMT), was inversely related to invasion capacity of glioma and to α5β1 integrin expression [116].

### 4.6. Melanoma

Malignant melanoma has a high metastatic potential. A role of α5β1 integrin in promoting melanoma metastasis through an increase in cell adhesion to fibronectin and protection against apoptosis was reported [117]. Recently, evidence that α5β1 integrin has a crucial role in melanoma metastasis confirmed this hypothesis. It was shown that α5 integrin subunit up-regulation was under the control of survivin [118] or controlled by the interaction between caveolin 1 and Rho-GTPases [119]. Curiously, the specific uveal melanoma seems to be one of the cases where α5β1 integrin expression negatively impacts on tumorigenicity. High aggressiveness of uveal melanoma cells is dependent on the loss of α5β1 integrin at the cell surface [120,121,122]. However, restoration of α5β1 integrin expression in high tumorigenic cells increased the cell resistance to stress *in vitro* and growth properties *in vivo* [121] which appears somewhat paradoxical. It has been proposed that the effect of α5β1 integrin on cell tumorigenicity depends on the endogenous expression of fibronectin by the tumoral cells.

## 5. Integrin α5β1 Antagonists

The search for specific α5β1 integrin antagonists has increased these last years. They are developed to understand the integrin pathophysiological behaviour in preclinical studies on endothelial and tumoral cells but also as therapeutic agents in the clinic [2]. As α5β1 integrin has been largely described as an unambiguous pro-angiogenic integrin, these antagonists are generally presented as potential anti-angiogenic agents. Three main classes of antagonists are described at the time, specific antibodies, small peptides or small non peptidic RGD-like molecules.

### 5.1. Antibodies

An α5β1 function-blocking murine antibody, IIA1, was used in preclinical studies [74,91]. It was able to inhibit *in vitro* invasion of ovarian tumor cells into Matrigel and tumor cell adhesion to mesothelial cells; it decreased the number and the size of intra-abdominal metastases and increased the survival of mice [74]. This inhibitory antibody also induced apoptosis of breast cancer cells in 3D culture conditions [91]. A chimeric humanised version of IIA1 antibody was generated, named volociximab (developed first by PDL Biopharma, Fremont, CA, USA), with similar affinity for α5β1 integrin and similar activity for blocking integrin adhesion to fibronectin than IIA1 [123]. Volociximab is a potent inhibitor of *in vitro* model of angiogenesis by inducing apoptosis of actively proliferating but not resting endothelial cells. It reduced vessel density and tumor growth in carcinoma xenografted in rabbits [123,124]. Results of volociximab in clinical assays have been reviewed recently in [125]. Volociximab has been shown to be safe and tolerable in phase I studies [126,127] in patients with different solid tumors. Reported adverse effects included constitutional symptoms, gastrointestinal symptoms, headache, edema and hypertension. Although α5β1 integrin is expressed on normal blood monocytes, no clinically apparent infectious complications were observed. A phase II clinical trial has shown that in patients with platinum-resistant advanced epithelial ovarian or primary peritoneal cancer, weekly monotherapy with volociximab was well tolerated but without efficacy on these particular population of patients [128]. In patients with refractory or relapsed metastasic clear cell renal carcinoma, volociximab led to stable disease in 80% of patients [129]. 

A dual functional monoclonal antibody, PF-04605412, has been developed by Pfizer. This antibody targets α5β1 integrin and was engineered to elicit potent antibody-dependent cellular toxicity [130]. Preclinical studies showed that PF-04605412 potently inhibited α5β1 integrin mediated intracellular signalling, cell adhesion, migration and angiogenesis. In animal studies, it displayed robust anti-tumor efficacy correlated with α5 integrin subunit expression, macrophages and natural killer cells infiltration [130]. A clinical trial phase I is currently underway in solid tumors refractory to available therapies.

### 5.2. RGD-like Molecules

The RGD motif of fibronectin is recognised by at least three main integrins: α5β1, αvβ3 and αIIbβ3. The challenge of these last ten years has been to design antagonists with enhanced selectivity for each of these integrins.

The first selective non peptidic antagonist for α5β1 integrin was SJ749 (compound 20 in [131]). SJ749 blocked efficiently α5 integrin-expressing HT29 cell adhesion to fibronectin and not to other ECM ligand. It also blocked α5β1 integrin function in chick embryo and murine models of angiogenesis acting as a potent inhibitor of tumor growth and tumor-induced angiogenesis [45]. We described that SJ749 potently inhibited the proliferation of glioma cell lines dependently of α5β1 integrin expression level [112,114] and that SJ749 sensitized glioma cells to chemotherapy by modulating the p53 pathway [113].

SJ749 was used in docking experiments to build a 3D model of the α5β1 integrin with the αvβ3 integrin crystal structure as a model [132]. Based on the characteristics of SJ749 binding site and SAR analysis, analogs of SJ749 [132] or original compounds [133,134,135] were designed by the group of H. Kessler (München, Germany) and tested for their integrin affinities. Compounds with high affinity and selectivity for α5β1 integrin were found by these strategies. Few data concerning the biological activities of such compounds are available to date. We evaluated the effects of one of these compounds, K34c, on glioma cell lines. We demonstrated that K34c affected the survival of glioma cells as well as their resistance to chemotherapies [110,113].

New selective small non peptidic α5β1 integrin antagonists were described by Jerini AG (Berlin, Germany) [136]. Compounds were mainly tested in pathological models of neovascularization where α5β1 integrin plays a crucial role [137,138,139,140,141]. One of them, JSM6427, was shown to attenuate glioma growth [141]. Interestingly, new orally available α5β1 integrin antagonists were described recently by this pharmaceutical group [142,143]. Other small non peptidic molecules were synthesized by AstraZeneca and showed some selectivity for α5β1 integrin compared to αvβ3 integrin [144,145].

### 5.3. Non RGD-like Peptides

Sequences outside of the RGD site are required to allow full adhesion of α5β1 integrin to fibronectin. Of particular interest is the sequence Pro-His-Ser-Arg-Asn (PHSRN) in the 9th type III repeat of fibronectin also called the “synergy site”. PHSRN peptide induced invasion of prostate tumor cells by inducing MMP-1 [146,147] and stimulation of angiogenesis [148] which was inhibited by the competitive inhibitor PHSCN peptide. The acetylated amidated PHSCN peptide was even more potent than PHSCN peptide [146], and was developed by Attenuon LLC (San Diego, CA, USA) under the name ATN-161. ATN-161 treatment blocks prostate tumor recurrence, metastasis and micrometastasis [149], reduces colorectal liver metastasis and improves survival when given in addition with chemotherapy [150], and blocks breast cancer growth and metastasis [151] in preclinical mouse models. Targeting α5β1 integrin with ATN-161 in combination with radiotherapy enhanced apoptosis of breast cancer cells grown in 3D culture [91]. ATN-161 proved also efficient to block choroidal neovascularisation [152]. Phase I trial of ATN-161 indicated that it was well tolerated in patients with solid tumors and that one third of patients manifested prolonged stable disease. No side effects emerged or became worse with continued chronic dosing of ATN-161 [153]. Recently, PHSCN dendrimers were synthesized and shown to be more potent than the initial peptide for inhibiting α5β1 integrin-mediated MMP-1 secretion *in vitro* and for inhibiting human prostate cancer cell invasion, extravasion and lung metastasis *in vivo* [154]. Similar results were reported on human breast cancer cells [155].

## 6. Conclusions

The critical role of α5β1 integrin in physiological angiogenesis and development has been recognized for over two decades. More recent are the data implicating α5β1 integrin in pathophysiological/tumoral neoangiogenesis. Even more recently, its role as a prognostic and diagnostic marker has been highlighted in several solid tumors. The relationship between high expression of α5β1 integrin in subpopulation of patients with solid tumor and a poor prognosis for these patients suggest its implication in resistance to conventional therapies. As shown above, α5β1 integrin is implicated in different aspects of tumor progression and appears particularly overexpressed in the most aggressive tumor grades. Ways to modulate positively the α5β1 integrin expression also appear multiple and certainly tissue dependent. Its participation in tumor angiogenesis and tumoral cell migration and adhesion to metastasis niches as well as its effects on therapy resistance make it a pertinent therapeutic target for the future. Several antagonists are being tested with some already reaching the clinic. Targeting α5β1 integrin appeared safe for the patients in the few clinical trials reported so far. To date, efforts have not focused on α5β1 integrin antagonists but data summarized here support the notion that they will play an increasing role in human therapy. The recent elucidation of the crystal structure of α5β1 integrin ectodomain will certainly help to define more potent and specific antagonists. The goal for the future will be to define clear molecular biomarkers to support the proposition of subpopulations of patients potentially sensitive to a targeted therapy against α5β1 integrin.

## References

[1] Desgrosellier J.S., Cheresh D.A. (2010). Integrins in cancer: Biological implications and therapeutic opportunities. Nat. Rev. Cancer.

[2] Goodman S.L., Picard M. (2012). Integrins as therapeutic targets. Trends Pharmacol. Sci..

[3] Aoudjit F., Vuori K. (2012). Integrin signaling in cancer cell survival and chemoresistance. Chemother. Res. Pract..

[4] Avraamides C.J., Garmy-Susini B., Varner J.A. (2008). Integrins in angiogenesis and lymphangiogenesis. Nat. Rev. Cancer.

[5] Chamberlain M.C., Cloughsey T., Reardon D.A., Wen P.Y. (2012). A novel treatment for glioblastoma: Integrin inhibition. Expert Rev. Neurother..

[6] Campbell I.D., Humphries M.J.  (2011). Integrin structure, activation, and interactions. Cold Spring Harb. Perspect. Biol..

[7] Kim C., Ye F., Ginsberg M.H. (2011). Regulation of integrin activation. Annu. Rev. Cell. Dev. Biol..

[8] Hu P., Luo B.H. (2013). Integrin bi-directional signaling across the plasma membrane. J. Cell. Physiol..

[9] Cox D., Brennan M., Moran N. (2010). Integrins as therapeutic targets: Lessons and opportunities. Nat. Rev. Drug Discov..

[10] Lee J.O., Bankston L.A., Arnaout M.A., Liddington R.C. (1995). Two conformations of the integrin A-domain (I-domain): A pathway for activation?. Structure.

[11] Aota S., Nomizu M., Yamada K.M. (1994). The short amino acid sequence Pro-His-Ser-Arg-Asn in human fibronectin enhances cell-adhesive function. J. Biol. Chem..

[12] Obara M., Kang M.S., Yamada K.M. (1988). Site-directed mutagenesis of the cell-binding domain of human fibronectin: Separable, synergistic sites mediate adhesive function. Cell.

[13] Nagae M., Re S., Mihara E., Nogi T., Sugita Y., Takagi J. (2012). Crystal structure of alpha5beta1 integrin ectodomain: Atomic details of the fibronectin receptor. J. Cell Biol..

[14] Barczyk M., Carracedo S., Gullberg D. (2010). Integrins. Cell Tissue Res..

[15] Xiong J.P., Stehle T., Diefenbach B., Zhang R., Dunker R., Scott D.L., Joachimiak A., Goodman S.L., Arnaout M.A. (2001). Crystal structure of the extracellular segment of integrin alpha Vbeta3. Science.

[16] Lau T.L., Kim C., Ginsberg M.H., Ulmer T.S. (2009). The structure of the integrin alphaIIbbeta3 transmembrane complex explains integrin transmembrane signalling. EMBO. J..

[17] Shattil S.J., Kim C., Ginsberg M.H. (2010). The final steps of integrin activation: The end game. Nat. Rev. Mol. Cell Biol..

[18] Pouwels J., Nevo J., Pellinen T., Ylanne J., Ivaska J. (2012). Negative regulators of integrin activity. J. Cell. Sci..

[19] Alahari S.K., Nasrallah H. (2004). A membrane proximal region of the integrin alpha5 subunit is important for its interaction with nischarin. Biochem. J..

[20] Valdembri D., Caswell P.T., Anderson K.I., Schwarz J.P., Konig I., Astanina E., Caccavari F., Norman J.C., Humphries M.J., Bussolino F. (2009). Neuropilin-1/GIPC1 signaling regulates alpha5beta1 integrin traffic and function in endothelial cells. PLoS Biol..

[21] Margadant C., Monsuur H.N., Norman J.C., Sonnenberg A. (2011). Mechanisms of integrin activation and trafficking. Curr. Opin. Cell Biol..

[22] Lobert V.H., Brech A., Pedersen N.M., Wesche J., Oppelt A., Malerod L., Stenmark H. (2010). Ubiquitination of alpha 5 beta 1 integrin controls fibroblast migration through lysosomal degradation of fibronectin-integrin complexes. Dev. Cell.

[23] Shi F., Sottile J. (2008). Caveolin-1-dependent beta1 integrin endocytosis is a critical regulator of fibronectin turnover. J. Cell. Sci..

[24] Pellinen T., Arjonen A., Vuoriluoto K., Kallio K., Fransen J.A., Ivaska J. (2006). Small GTPase Rab21 regulates cell adhesion and controls endosomal traffic of beta1-integrins. J. Cell Biol..

[25] Caswell P.T., Chan M., Lindsay A.J., McCaffrey M.W., Boettiger D., Norman J.C. (2008). Rab-coupling protein coordinates recycling of alpha5beta1 integrin and EGFR1 to promote cell migration in 3D microenvironments. J. Cell Biol..

[26] Bridgewater R.E., Norman J.C., Caswell P.T. (2012). Integrin trafficking at a glance. J. Cell. Sci..

[27] Caswell P.T., Spence H.J., Parsons M., White D.P., Clark K., Cheng K.W., Mills G.B., Humphries M.J., Messent A.J., Anderson K.I. (2007). Rab25 associates with alpha5beta1 integrin to promote invasive migration in 3D microenvironments. Dev. Cell.

[28] Schwartz M.A. (2010). Integrins and extracellular matrix in mechanotransduction. Cold Spring Harb. Perspect. Biol..

[29] Roca-Cusachs P., Iskratsch T., Sheetz M.P. (2012). Finding the weakest link: Exploring integrin-mediated mechanical molecular pathways. J. Cell. Sci..

[30] Roca-Cusachs P., Gauthier N.C., Del Rio A., Sheetz M.P. (2009). Clustering of alpha(5)beta(1) integrins determines adhesion strength whereas alpha(v)beta(3) and talin enable mechanotransduction. Proc. Natl. Acad. Sci. USA.

[31] Friedland J.C., Lee M.H., Boettiger D. (2009). Mechanically activated integrin switch controls alpha5beta1 function. Science.

[32] Mierke C.T., Frey B., Fellner M., Herrmann M., Fabry B. (2011). Integrin alpha5beta1 facilitates cancer cell invasion through enhanced contractile forces. J. Cell. Sci..

[33] Menon S., Beningo K.A. (2011). Cancer cell invasion is enhanced by applied mechanical stimulation. PLoS One.

[34] Yang J.T., Rayburn H., Hynes R.O. (1995). Cell adhesion events mediated by alpha 4 integrins are essential in placental and cardiac development. Development.

[35] Bader B.L., Rayburn H., Crowley D., Hynes R.O. (1998). Extensive vasculogenesis, angiogenesis, and organogenesis precede lethality in mice lacking all alpha v integrins. Cell.

[36] Carlson T.R., Hu H., Braren R., Kim Y.H., Wang R.A. (2008). Cell-autonomous requirement for beta1 integrin in endothelial cell adhesion, migration and survival during angiogenesis in mice. Development.

[37] Yang J.T., Rayburn H., Hynes R.O. (1993). Embryonic mesodermal defects in alpha 5 integrin-deficient mice. Development.

[38] Goh K.L., Yang J.T., Hynes R.O. (1997). Mesodermal defects and cranial neural crest apoptosis in alpha5 integrin-null embryos. Development.

[39] George E.L., Georges-Labouesse E.N., Patel-King R.S., Rayburn H., Hynes R.O. (1993). Defects in mesoderm, neural tube and vascular development in mouse embryos lacking fibronectin. Development.

[40] Van der Flier A., Badu-Nkansah K., Whittaker C.A., Crowley D., Bronson R.T., Lacy-Hulbert A., Hynes R.O. (2010). Endothelial alpha5 and alphav integrins cooperate in remodeling of the vasculature during development. Development.

[41] Li L., Welser-Alves J., van der Flier A., Boroujerdi A., Hynes R.O., Milner R. (2012). An angiogenic role for the alpha5beta1 integrin in promoting endothelial cell proliferation during cerebral hypoxia. Exp. Neurol..

[42] Sudhakar A., Sugimoto H., Yang C., Lively J., Zeisberg M., Kalluri R. (2003). Human tumstatin and human endostatin exhibit distinct antiangiogenic activities mediated by alpha v beta 3 and alpha 5 beta 1 integrins. Proc. Natl. Acad. Sci. USA.

[43] Orecchia A., Lacal P.M., Schietroma C., Morea V., Zambruno G., Failla C.M. (2003). Vascular endothelial growth factor receptor-1 is deposited in the extracellular matrix by endothelial cells and is a ligand for the alpha 5 beta 1 integrin. J. Cell. Sci..

[44] Felcht M., Luck R., Schering A., Seidel P., Srivastava K., Hu J., Bartol A., Kienast Y., Vettel C., Loos E.K. (2012). Angiopoietin-2 differentially regulates angiogenesis through TIE2 and integrin signaling. J. Clin. Invest..

[45] Kim S., Bell K., Mousa S.A., Varner J.A. (2000). Regulation of angiogenesis *in vivo* by ligation of integrin alpha5beta1 with the central cell-binding domain of fibronectin. Am. J. Pathol..

[46] Magnussen A., Kasman I.M., Norberg S., Baluk P., Murray R., McDonald D.M. (2005). Rapid access of antibodies to alpha5beta1 integrin overexpressed on the luminal surface of tumor blood vessels. Cancer Res..

[47] Zhang H., Li C., Baciu P.C. (2002). Expression of integrins and MMPs during alkaline-burn-induced corneal angiogenesis. Invest. Ophthalmol. Vis. Sci..

[48] Bussolati B., Deambrosis I., Russo S., Deregibus M.C., Camussi G. (2003). Altered angiogenesis and survival in human tumor-derived endothelial cells. FASEB J..

[49] Parsons-Wingerter P., Kasman I.M., Norberg S., Magnussen A., Zanivan S., Rissone A., Baluk P., Favre C.J., Jeffry U., Murray R. (2005). Uniform overexpression and rapid accessibility of alpha5beta1 integrin on blood vessels in tumors. Am. J. Pathol..

[50] Klein S., de Fougerolles A.R., Blaikie P., Khan L., Pepe A., Green C.D., Koteliansky V., Giancotti F.G. (2002). Alpha 5 beta 1 integrin activates an NF-kappa B-dependent program of gene expression important for angiogenesis and inflammation. Mol. Cell. Biol..

[51] Kim S., Bakre M., Yin H., Varner J.A. (2002). Inhibition of endothelial cell survival and angiogenesis by protein kinase A. J. Clin. Invest..

[52] Stupack D.G., Puente X.S., Boutsaboualoy S., Storgard C.M., Cheresh D.A. (2001). Apoptosis of adherent cells by recruitment of caspase-8 to unligated integrins. J. Cell. Biol..

[53] Bhaskar V., Zhang D., Fox M., Seto P., Wong M.H., Wales P.E., Powers D., Chao D.T., Dubridge R.B., Ramakrishnan V. (2007). A function blocking anti-mouse integrin alpha5beta1 antibody inhibits angiogenesis and impedes tumor growth *in vivo*. J. Transl. Med..

[54] Varner J.A., Emerson D.A., Juliano R.L. (1995). Integrin alpha 5 beta 1 expression negatively regulates cell growth: Reversal by attachment to fibronectin. Mol. Biol. Cell..

[55] O'Brien V., Frisch S.M., Juliano R.L. (1996). Expression of the integrin alpha 5 subunit in HT29 colon carcinoma cells suppresses apoptosis triggered by serum deprivation. Exp. Cell. Res..

[56] Schmidt R., Streit M., Kaiser R., Herzberg F., Schirner M., Schramm K., Kaufmann C., Henneken M., Schafer-Korting M., Thiel E. (1998). *De novo* expression of the alpha5beta1-fibronectin receptor in HT29 colon-cancer cells reduces activity of C-SRC. Increase of C-SRC activity by attachment on fibronectin. Int. J. Cancer.

[57] Schirner M., Herzberg F., Schmidt R., Streit M., Schoning M., Hummel M., Kaufmann C., Thiel E., Kreuser E.D. (1998). Integrin alpha5beta1: A potent inhibitor of experimental lung metastasis. Clin. Exp. Metastasis.

[58] Chantret I., Barbat A., Dussaulx E., Brattain M.G., Zweibaum A. (1988). Epithelial polarity, villin expression, and enterocytic differentiation of cultured human colon carcinoma cells: A survey of twenty cell lines. Cancer Res..

[59] Gong J., Wang D., Sun L., Zborowska E., Willson J.K., Brattain M.G. (1997). Role of alpha 5 beta 1 integrin in determining malignant properties of colon carcinoma cells. Cell Growth Differ..

[60] Murillo C.A., Rychahou P.G., Evers B.M. (2004). Inhibition of alpha5 integrin decreases PI3K activation and cell adhesion of human colon cancers. Surgery.

[61] Toquet C., Colson A., Jarry A., Bezieau S., Volteau C., Boisseau P., Merlin D., Laboisse C.L., Mosnier J.F. (2012). ADAM15 to alpha5beta1 integrin switch in colon carcinoma cells: A late event in cancer progression associated with tumor dedifferentiation and poor prognosis. Int. J. Cancer.

[62] Koike T., Kimura N., Miyazaki K., Yabuta T., Kumamoto K., Takenoshita S., Chen J., Kobayashi M., Hosokawa M., Taniguchi A. (2004). Hypoxia induces adhesion molecules on cancer cells: A missing link between Warburg effect and induction of selectin-ligand carbohydrates. Proc. Natl. Acad. Sci. USA.

[63] Anderson J.A., Grabowska A.M., Watson S.A. (2007). PTHrP increases transcriptional activity of the integrin subunit alpha5. Br. J. Cancer.

[64] Nam E.H., Lee Y., Park Y.K., Lee J.W., Kim S. (2012). ZEB2 upregulates integrin alpha5 expression through cooperation with Sp1 to induce invasion during epithelial-mesenchymal transition of human cancer cells. Carcinogenesis.

[65] Reyes-Reyes M.E., George M.D., Roberts J.D., Akiyama S.K. (2006). P-selectin activates integrin-mediated colon carcinoma cell adhesion to fibronectin. Exp. Cell. Res..

[66] Camacho-Leal P., Zhai A.B., Stanners C.P. (2007). A co-clustering model involving alpha5beta1 integrin for the biological effects of GPI-anchored human carcinoembryonic antigen (CEA). J. Cell. Physiol..

[67] Dia V.P., Mejia E.G. (2011). Lunasin promotes apoptosis in human colon cancer cells by mitochondrial pathway activation and induction of nuclear clusterin expression. Cancer Lett..

[68] Shibata K., Kikkawa F., Nawa A., Suganuma N., Hamaguchi M. (1997). Fibronectin secretion from human peritoneal tissue induces Mr 92,000 type IV collagenase expression and invasion in ovarian cancer cell lines. Cancer Res..

[69] Casey R.C., Burleson K.M., Skubitz K.M., Pambuccian S.E., Oegema T.R., Ruff L.E., Skubitz A.P. (2001). Beta 1-integrins regulate the formation and adhesion of ovarian carcinoma multicellular spheroids. Am. J. Pathol..

[70] Burleson K.M., Casey R.C., Skubitz K.M., Pambuccian S.E., Oegema T.R., Skubitz A.P. (2004). Ovarian carcinoma ascites spheroids adhere to extracellular matrix components and mesothelial cell monolayers. Gynecol. Oncol..

[71] Yokoyama Y., Ramakrishnan S. (2007). Binding of endostatin to human ovarian cancer cells inhibits cell attachment. Int. J. Cancer.

[72] Yokoyama Y., Sedgewick G., Ramakrishnan S. (2007). Endostatin binding to ovarian cancer cells inhibits peritoneal attachment and dissemination. Cancer Res..

[73] Dong Y., Tan O.L., Loessner D., Stephens C., Walpole C., Boyle G.M., Parsons P.G., Clements J.A. (2010). Kallikrein-related peptidase 7 promotes multicellular aggregation via the alpha(5)beta(1) integrin pathway and paclitaxel chemoresistance in serous epithelial ovarian carcinoma. Cancer Res..

[74] Sawada K., Mitra A.K., Radjabi A.R., Bhaskar V., Kistner E.O., Tretiakova M., Jagadeeswaran S., Montag A., Becker A., Kenny H.A. (2008). Loss of E-cadherin promotes ovarian cancer metastasis via alpha 5-integrin, which is a therapeutic target. Cancer Res..

[75] Li Q., Liu S., Lin B., Yan L., Wang Y., Wang C., Zhang S. (2010). Expression and correlation of Lewis y antigen and integrins alpha5 and beta1 in ovarian serous and mucinous carcinoma. Int. J. Gynecol. Cancer.

[76] Mitra A.K., Sawada K., Tiwari P., Mui K., Gwin K., Lengyel E. (2011). Ligand-independent activation of c-Met by fibronectin and alpha(5)beta(1)-integrin regulates ovarian cancer invasion and metastasis. Oncogene.

[77] Deng B., Zhang S., Miao Y., Han Z., Zhang X., Wen F., Zhang Y. (2012). Adrenomedullin expression in epithelial ovarian cancers and promotes HO8910 cell migration associated with upregulating integrin alpha5beta1 and phosphorylating FAK and paxillin. J. Exp. Clin. Cancer Res..

[78] Sawada K., Ohyagi-Hara C., Kimura T., Morishige K. (2012). Integrin inhibitors as a therapeutic agent for ovarian cancer. J. Oncol..

[79] Seftor R.E., Seftor E.A., Sheng S., Pemberton P.A., Sager R., Hendrix M.J. (1998). Maspin suppresses the invasive phenotype of human breast carcinoma. Cancer Res..

[80] Ignatoski K.M., Maehama T., Markwart S.M., Dixon J.E., Livant D.L., Ethier S.P. (2000). ERBB-2 overexpression confers PI 3' kinase-dependent invasion capacity on human mammary epithelial cells. Br. J. Cancer.

[81] Jia Y., Zeng Z.Z., Markwart S.M., Rockwood K.F., Ignatoski K.M., Ethier S.P., Livant D.L. (2004). Integrin fibronectin receptors in matrix metalloproteinase-1-dependent invasion by breast cancer and mammary epithelial cells. Cancer Res..

[82] Maschler S., Wirl G., Spring H., Bredow D.V., Sordat I., Beug H., Reichmann E. (2005). Tumor cell invasiveness correlates with changes in integrin expression and localization. Oncogene.

[83] Spangenberg C., Lausch E.U., Trost T.M., Prawitt D., May A., Keppler R., Fees S.A., Reutzel D., Bell C., Schmitt S. (2006). ERBB2-mediated transcriptional up-regulation of the alpha5beta1 integrin fibronectin receptor promotes tumor cell survival under adverse conditions. Cancer Res..

[84] Qin L., Chen X., Wu Y., Feng Z., He T., Wang L., Liao L., Xu J. (2011). Steroid receptor coactivator-1 upregulates integrin alpha(5) expression to promote breast cancer cell adhesion and migration. Cancer Res..

[85] Baranwal S., Wang Y., Rathinam R., Lee J., Jin L., McGoey R., Pylayeva Y., Giancotti F., Blobe G.C., Alahari S.K. (2011). Molecular characterization of the tumor-suppressive function of nischarin in breast cancer. J. Natl. Cancer Inst..

[86] Wong A.W., Paulson Q.X., Hong J., Stubbins R.E., Poh K., Schrader E., Nunez N.P. (2011). Alcohol promotes breast cancer cell invasion by regulating the Nm23-ITGA5 pathway. J. Exp. Clin. Cancer Res..

[87] Wu H., Liang Y.L., Li Z., Jin J., Zhang W., Duan L., Zha X. (2006). Positive expression of E-cadherin suppresses cell adhesion to fibronectin via reduction of alpha5beta1 integrin in human breast carcinoma cells. J. Cancer Res. Clin. Oncol..

[88] Imanishi Y., Hu B., Jarzynka M.J., Guo P., Elishaev E., Bar-Joseph I., Cheng S.Y. (2007). Angiopoietin-2 stimulates breast cancer metastasis through the alpha(5)beta(1) integrin-mediated pathway. Cancer Res..

[89] Morozevich G., Kozlova N., Cheglakov I., Ushakova N., Berman A. (2009). Integrin alpha5beta1 controls invasion of human breast carcinoma cells by direct and indirect modulation of MMP-2 collagenase activity. Cell Cycle.

[90] Baranwal S., Wang Y., Rathinam R., Lee J., Jin L., McGoey R., Pylayeva Y., Giancotti F., Blobe G.C., Alahari S.K. (2011). Molecular characterization of the tumor-suppressive function of nischarin in breast cancer. J. Natl. Cancer Inst..

[91] Nam J.M., Onodera Y., Bissell M.J., Park C.C. (2010). Breast cancer cells in three-dimensional culture display an enhanced radioresponse after coordinate targeting of integrin alpha5beta1 and fibronectin. Cancer Res..

[92] Mythreye K., Knelson E.H., Gatza C.E., Gatza M.L., Blobe G.C. (2012). TbetaRIII/beta-arrestin2 regulates integrin alpha5beta1 trafficking, function, and localization in epithelial cells. Oncogene.

[93] Wang Y., Shenouda S., Baranwal S., Rathinam R., Jain P., Bao L., Hazari S., Dash S., Alahari S.K. (2011). Integrin subunits alpha5 and alpha6 regulate cell cycle by modulating the chk1 and Rb/E2F pathways to affect breast cancer metastasis. Mol. Cancer.

[94] Dingemans A.M., van den Boogaart V., Vosse B.A., van Suylen R.J., Griffioen A.W., Thijssen V.L. (2010). Integrin expression profiling identifies integrin alpha5 and beta1 as prognostic factors in early stage non-small cell lung cancer. Mol. Cancer.

[95] Adachi M., Taki T., Higashiyama M., Kohno N., Inufusa H., Miyake M. (2000). Significance of integrin alpha5 gene expression as a prognostic factor in node-negative non-small cell lung cancer. Clin. Cancer Res..

[96] Han J.Y., Kim H.S., Lee S.H., Park W.S., Lee J.Y., Yoo N.J. (2003). Immunohistochemical expression of integrins and extracellular matrix proteins in non-small cell lung cancer: Correlation with lymph node metastasis. Lung Cancer.

[97] Lawson M.H., Cummings N.M., Rassl D.M., Vowler S.L., Wickens M., Howat W.J., Brenton J.D., Murphy G., Rintoul R.C. (2010). Bcl-2 and beta1-integrin predict survival in a tissue microarray of small cell lung cancer. Br. J. Cancer.

[98] Roman J., Ritzenthaler J.D., Roser-Page S., Sun X., Han S. (2010). alpha5beta1-integrin expression is essential for tumor progression in experimental lung cancer. Am. J. Respir. Cell Mol. Biol..

[99] Han S., Khuri F.R., Roman J. (2006). Fibronectin stimulates non-small cell lung carcinoma cell growth through activation of Akt/mammalian target of rapamycin/S6 kinase and inactivation of LKB1/AMP-activated protein kinase signal pathways. Cancer Res..

[100] Ritzenthaler J.D., Han S., Roman J. (2008). Stimulation of lung carcinoma cell growth by fibronectin-integrin signalling. Mol. Biosyst..

[101] Caccavari F., Valdembri D., Sandri C., Bussolino F., Serini G. (2009). Integrin signaling and lung cancer. Cell Adh. Migr..

[102] Sethi T., Rintoul R.C., Moore S.M., MacKinnon A.C., Salter D., Choo C., Chilvers E.R., Dransfield I., Donnelly S.C., Strieter R., Haslett C. (1999). Extracellular matrix proteins protect small cell lung cancer cells against apoptosis: A mechanism for small cell lung cancer growth and drug resistance *in vivo*. Nat. Med..

[103] Rintoul R.C., Sethi T. (2002). Extracellular matrix regulation of drug resistance in small-cell lung cancer. Clin. Sci. (Lond.).

[104] Buttery R.C., Rintoul R.C., Sethi T. (2004). Small cell lung cancer: The importance of the extracellular matrix. Int. J. Biochem. Cell Biol..

[105] Hodkinson P.S., Elliott T., Wong W.S., Rintoul R.C., Mackinnon A.C., Haslett C., Sethi T. (2006). ECM overrides DNA damage-induced cell cycle arrest and apoptosis in small-cell lung cancer cells through beta1 integrin-dependent activation of PI3-kinase. Cell Death Differ..

[106] Gingras M.C., Roussel E., Bruner J.M., Branch C.D., Moser R.P. (1995). Comparison of cell adhesion molecule expression between glioblastoma multiforme and autologous normal brain tissue. J. Neuroimmunol..

[107] Riemenschneider M.J., Mueller W., Betensky R.A., Mohapatra G., Louis D.N. (2005). *In situ* analysis of integrin and growth factor receptor signaling pathways in human glioblastomas suggests overlapping relationships with focal adhesion kinase activation. Am. J. Pathol..

[108] Kita D., Takino T., Nakada M., Takahashi T., Yamashita J., Sato H. (2001). Expression of dominant-negative form of Ets-1 suppresses fibronectin-stimulated cell adhesion and migration through down-regulation of integrin alpha5 expression in U251 glioma cell line. Cancer Res..

[109] Janouskova H., Maglott A., Leger D.Y., Bossert C., Noulet F., Guerin E., Guenot D., Pinel S., Chastagner P., Plenat F. (2012). Integrin alpha5beta1 plays a critical role in resistance to temozolomide by interfering with the p53 pathway in high-grade glioma. Cancer Res..

[110] Cosset E.C., Godet J., Entz-Werle N., Guerin E., Guenot D., Froelich S., Bonnet D., Pinel S., Plenat F., Chastagner P. (2012). Involvement of the TGFbeta pathway in the regulation of alpha5 beta1 integrins by caveolin-1 in human glioblastoma. Int. J. Cancer.

[111] Holmes K.M., Annala M., Chua C.Y., Dunlap S.M., Liu Y., Hugen N., Moore L.M., Cogdell D., Hu L., Nykter M. (2012). Insulin-like growth factor-binding protein 2-driven glioma progression is prevented by blocking a clinically significant integrin, integrin-linked kinase, and NF-kappaB network. Proc. Natl. Acad. Sci. USA.

[112] Martin S., Cosset E.C., Terrand J., Maglott A., Takeda K., Dontenwill M. (2009). Caveolin-1 regulates glioblastoma aggressiveness through the control of alpha(5)beta(1) integrin expression and modulates glioblastoma responsiveness to SJ749, an alpha(5)beta(1) integrin antagonist. Biochim. Biophys. Acta.

[113] Martinkova E., Maglott A., Leger D.Y., Bonnet D., Stiborova M., Takeda K., Martin S., Dontenwill M. (2010). alpha5beta1 integrin antagonists reduce chemotherapy-induced premature senescence and facilitate apoptosis in human glioblastoma cells. Int. J. Cancer.

[114] Maglott A., Bartik P., Cosgun S., Klotz P., Ronde P., Fuhrmann G., Takeda K., Martin S., Dontenwill M. (2006). The small alpha5beta1 integrin antagonist, SJ749, reduces proliferation and clonogenicity of human astrocytoma cells. Cancer Res..

[115] Kesanakurti D., Chetty C., Dinh D.H., Gujrati M., Rao J.S. (2012). Role of MMP-2 in the regulation of IL-6/Stat3 survival signaling via interaction with alpha5beta1 integrin in glioma. Oncogene.

[116] Chahal M., Abdulkarim B., Xu Y., Guiot M.C., Easaw J.C., Stifani N., Sabri S. (2012). O(6)-Methylguanine-DNA Methyltransferase Is a Novel Negative Effector of Invasion in Glioblastoma Multiforme. Mol. Cancer Ther..

[117] Qian F., Zhang Z.C., Wu X.F., Li Y.P., Xu Q. (2005). Interaction between integrin alpha(5) and fibronectin is required for metastasis of B16F10 melanoma cells. Biochem. Biophys. Res. Commun..

[118] McKenzie J.A., Liu T., Goodson A.G., Grossman D. (2010). Survivin enhances motility of melanoma cells by supporting Akt activation and {alpha}5 integrin upregulation. Cancer Res..

[119] Arpaia E., Blaser H., Quintela-Fandino M., Duncan G., Leong H.S., Ablack A., Nambiar S.C., Lind E.F., Silvester J., Fleming C.K. (2012). The interaction between caveolin-1 and Rho-GTPases promotes metastasis by controlling the expression of alpha5-integrin and the activation of Src, Ras and Erk. Oncogene.

[120] Beliveau A., Berube M., Rousseau A., Pelletier G., Guerin S.L. (2000). Expression of integrin alpha5beta1 and MMPs associated with epithelioid morphology and malignancy of uveal melanoma. Invest. Ophthalmol. Vis. Sci..

[121] Beliveau A., Berube M., Carrier P., Mercier C., Guerin S.L. (2001). Tumorigenicity of the mixed spindle-epithelioid SP6. 5 and epithelioid TP17 uveal melanoma cell lines is differentially related to alpha5beta1 integrin expression. Invest. Ophthalmol. Vis. Sci..

[122] Landreville S., Vigneault F., Bergeron M.A., Leclerc S., Gaudreault M., Morcos M., Mouriaux F., Salesse C., Guerin S.L. (2011). Suppression of alpha5 gene expression is closely related to the tumorigenic properties of uveal melanoma cell lines. Pigment. Cell Melanoma Res..

[123] Ramakrishnan V., Bhaskar V., Law D.A., Wong M.H., DuBridge R.B., Breinberg D., O'Hara C., Powers D.B., Liu G., Grove J. (2006). Preclinical evaluation of an anti-alpha5beta1 integrin antibody as a novel anti-angiogenic agent. J. Exp. Ther. Oncol..

[124] Bhaskar V., Fox M., Breinberg D., Wong M.H., Wales P.E., Rhodes S., DuBridge R.B., Ramakrishnan V. (2008). Volociximab, a chimeric integrin alpha5beta1 antibody, inhibits the growth of VX2 tumors in rabbits. Invest. New Drugs.

[125] Almokadem S., Belani C.P. (2011). Volociximab in cancer. Expert Opin. Biol. Ther..

[126] Besse B., Tsao L.C., Chao D.T., Fang Y., Soria J.C., Almokadem S., Belani C.P.  (2012). Phase Ib safety and pharmacokinetic study of volociximab, an anti-alpha5beta1 integrin antibody, in combination with carboplatin and paclitaxel in advanced non-small-cell lung cancer. Ann. Oncol..

[127] Ricart A.D., Tolcher A.W., Liu G., Holen K., Schwartz G., Albertini M., Weiss G., Yazji S., Ng C., Wilding G. (2008). Volociximab, a chimeric monoclonal antibody that specifically binds alpha5beta1 integrin: A phase I, pharmacokinetic, and biological correlative study. Clin. Cancer Res..

[128] Bell-McGuinn K.M., Matthews C.M., Ho S.N., Barve M., Gilbert L., Penson R.T., Lengyel E., Palaparthy R., Gilder K., Vassos A. (2011). A phase II, single-arm study of the anti-alpha5beta1 integrin antibody volociximab as monotherapy in patients with platinum-resistant advanced epithelial ovarian or primary peritoneal cancer. Gynecol. Oncol..

[129] Yazji S., Bukowski R., Kondagunta V., Figlin R. (2007). Final results from phase II study of volociximab, an α5β1 anti-integrin antibody, in refractory or relapsed metastatic clear cell renal cell carcinoma (mCCRCC). J. Clin. Oncol..

[130] Li G., Zhang L., Chen E., Wang J., Jiang X., Chen J.H., Wickman G., Amundson K., Bergqvist S., Zobel J. (2010). Dual functional monoclonal antibody PF-04605412 targets integrin alpha5beta1 and elicits potent antibody-dependent cellular cytotoxicity. Cancer Res..

[131] Smallheer J.M., Weigelt C.A., Woerner F.J., Wells J.S., Daneker W.F., Mousa S.A., Wexler R.R., Jadhav P.K. (2004). Synthesis and biological evaluation of nonpeptide integrin antagonists containing spirocyclic scaffolds. Bioorg. Med. Chem. Lett..

[132] Marinelli L., Meyer A., Heckmann D., Lavecchia A., Novellino E., Kessler H. (2005). Ligand binding analysis for human alpha5beta1 integrin: Strategies for designing new alpha5beta1 integrin antagonists. J. Med. Chem..

[133] Heckmann D., Meyer A., Marinelli L., Zahn G., Stragies R., Kessler H. (2007). Probing integrin selectivity: Rational design of highly active and selective ligands for the alpha5beta1 and alphavbeta3 integrin receptor. Angew. Chem. Int. Ed. Engl..

[134] Heckmann D., Meyer A., Laufer B., Zahn G., Stragies R., Kessler H. (2008). Rational design of highly active and selective ligands for the alpha5beta1 integrin receptor. Chembiochem.

[135] Meyer A., Auernheimer J., Modlinger A., Kessler H. (2006). Targeting RGD recognizing integrins: Drug development, biomaterial research, tumor imaging and targeting. Curr. Pharm. Des..

[136] Stragies R., Osterkamp F., Zischinsky G., Vossmeyer D., Kalkhof H., Reimer U., Zahn G. (2007). Design and synthesis of a new class of selective integrin alpha5beta1 antagonists. J. Med. Chem..

[137] Umeda N., Kachi S., Akiyama H., Zahn G., Vossmeyer D., Stragies R., Campochiaro P.A. (2006). Suppression and regression of choroidal neovascularization by systemic administration of an alpha5beta1 integrin antagonist. Mol. Pharmacol..

[138] Muether P.S., Dell S., Kociok N., Zahn G., Stragies R., Vossmeyer D., Joussen A.M. (2007). The role of integrin alpha5beta1 in the regulation of corneal neovascularization. Exp. Eye Res..

[139] Zahn G., Vossmeyer D., Stragies R., Wills M., Wong C.G., Loffler K.U., Adamis A.P., Knolle J. (2009). Preclinical evaluation of the novel small-molecule integrin alpha5beta1 inhibitor JSM6427 in monkey and rabbit models of choroidal neovascularization. Arch. Ophthalmol..

[140] Zahn G., Volk K., Lewis G.P., Vossmeyer D., Stragies R., Heier J.S., Daniel P.E., Adamis A.P., Chapin E.A., Fisher S.K. (2010). Assessment of the integrin alpha5beta1 antagonist JSM6427 in proliferative vitreoretinopathy using *in vitro* assays and a rabbit model of retinal detachment. Invest. Ophthalmol. Vis. Sci..

[141] Okazaki T., Ni A., Ayeni O.A., Baluk P., Yao L.C., Vossmeyer D., Zischinsky G., Zahn G., Knolle J., Christner C., McDonald D.M. (2009). alpha5beta1 Integrin blockade inhibits lymphangiogenesis in airway inflammation. Am. J. Pathol..

[142] Zischinsky G., Osterkamp F., Vossmeyer D., Zahn G., Scharn D., Zwintscher A., Stragies R. (2010). SAR of *N*-phenyl piperidine based oral integrin alpha5beta1 antagonists. Bioorg. Med. Chem. Lett..

[143] Zischinsky G., Osterkamp F., Vossmeyer D., Zahn G., Scharn D., Zwintscher A., Stragies R. (2010). Discovery of orally available integrin alpha5beta1 antagonists. Bioorg. Med. Chem. Lett..

[144] Delouvrie B., Al-Kadhimi K., Arnould J.C., Barry S.T., Cross D.A., Didelot M., Gavine P.R., Germain H., Harris C.S., Hughes A.M. (2012). Structure-activity relationship of a series of non peptidic RGD integrin antagonists targeting alpha5beta1: Part 1. Bioorg. Med. Chem. Lett..

[145] Delouvrie B., Al-Kadhimi K., Arnould J.C., Barry S.T., Cross D.A., Didelot M., Gavine P.R., Germain H., Harris C.S., Hughes A.M. (2012). Structure-activity relationship of a series of non peptidic RGD integrin antagonists targeting alpha5beta1: Part 2. Bioorg. Med. Chem. Lett..

[146] Livant D.L., Brabec R.K., Pienta K.J., Allen D.L., Kurachi K., Markwart S., Upadhyaya A. (2000). Anti-invasive, antitumorigenic, and antimetastatic activities of the PHSCN sequence in prostate carcinoma. Cancer Res..

[147] Livant D.L., Brabec R.K., Kurachi K., Allen D.L., Wu Y., Haaseth R., Andrews P., Ethier S.P., Markwart S. (2000). The PHSRN sequence induces extracellular matrix invasion and accelerates wound healing in obese diabetic mice. J. Clin. Invest..

[148] Zeng Z.Z., Yao H., Staszewski E.D., Rockwood K.F., Markwart S.M., Fay K.S., Spalding A.C., Livant D.L. (2009). alpha(5)beta(1) Integrin Ligand PHSRN Induces Invasion and alpha(5) mRNA in Endothelial Cells to Stimulate Angiogenesis. Transl. Oncol..

[149] Van Golen K.L., Bao L., Brewer G.J., Pienta K.J., Kamradt J.M., Livant D.L., Merajver S.D. (2002). Suppression of tumor recurrence and metastasis by a combination of the PHSCN sequence and the antiangiogenic compound tetrathiomolybdate in prostate carcinoma. Neoplasia.

[150] Stoeltzing O., Liu W., Reinmuth N., Fan F., Parry G.C., Parikh A.A., McCarty M.F., Bucana C.D., Mazar A.P., Ellis L.M. (2003). Inhibition of integrin alpha5beta1 function with a small peptide (ATN-161) plus continuous 5-FU infusion reduces colorectal liver metastases and improves survival in mice. Int. J. Cancer.

[151] Khalili P., Arakelian A., Chen G., Plunkett M.L., Beck I., Parry G.C., Donate F., Shaw D.E., Mazar A.P., Rabbani S.A. (2006). A non-RGD-based integrin binding peptide (ATN-161) blocks breast cancer growth and metastasis *in vivo*. Mol. Cancer Ther..

[152] Wang W., Wang F., Lu F., Xu S., Hu W., Huang J., Gu Q., Sun X. (2011). The antiangiogenic effects of integrin alpha5beta1 inhibitor (ATN-161) *in vitro* and *in vivo*. Invest. Ophthalmol. Vis. Sci..

[153] Cianfrocca M.E., Kimmel K.A., Gallo J., Cardoso T., Brown M.M., Hudes G., Lewis N., Weiner L., Lam G.N., Brown S.C. (2006). Phase 1 trial of the antiangiogenic peptide ATN-161 (Ac-PHSCN-NH(2)), a beta integrin antagonist, in patients with solid tumours. Br. J. Cancer.

[154] Yao H., Veine D.M., Zeng Z.Z., Fay K.S., Staszewski E.D., Livant D.L. (2010). Increased potency of the PHSCN dendrimer as an inhibitor of human prostate cancer cell invasion, extravasation, and lung colony formation. Clin. Exp. Metastasis.

[155] Yao H., Veine D.M., Fay K.S., Staszewski E.D., Zeng Z.Z., Livant D.L. (2011). The PHSCN dendrimer as a more potent inhibitor of human breast cancer cell invasion, extravasation, and lung colony formation. Breast Cancer Res. Treat..

